# The Role of Copper (II) on Kininogen Binding to Tropomyosin in the Presence of a Histidine–Proline-Rich Peptide

**DOI:** 10.3390/ijms21249343

**Published:** 2020-12-08

**Authors:** Anna Maria Santoro, Stefania Zimbone, Antonio Magrì, Diego La Mendola, Giulia Grasso

**Affiliations:** 1 CNR Istituto di Cristallografia Sede Secondaria di Catania, Via Gaifami 18, 95126 Catania, Italy; annamaria.santoro@cnr.it (A.M.S.); stefania_zimbone@libero.it (S.Z.); leotony@unict.it (A.M.); 2 Dipartimento di Farmacia, Università di Pisa, Via Bonanno Pisano 6, 56126 Pisa, Italy

**Keywords:** tropomyosin, kininogen, copper, zinc, angiogenesis, histidine–proline-rich glycoprotein, circular dichroism

## Abstract

The antiangiogenic activity of the H/P domain of histidine–proline-rich glycoprotein is mediated by its binding with tropomyosin, a protein exposed on endothelial cell-surface during the angiogenic switch, in presence of zinc ions. Although it is known that copper ion serum concentration is significantly increased in cancer patients, its role in the interaction of H/P domain with tropomyosin, has not yet been studied. In this paper, by using ELISA assay, we determined the modulating effect of TetraHPRG peptide, a sequence of 20 aa belonging to H/P domain, on the binding of Kininogen (HKa) with tropomyosin, both in absence and presence of copper and zinc ions. A potentiometric study was carried out to characterize the binding mode adopted by metal ions with TetraHPRG, showing the formation of complex species involving imidazole amide nitrogen atoms in metal binding. Moreover, circular dichroism showed a conformational modification of ternary systems formed by TetraHPRG, HKa and copper or zinc. Interestingly, slight pH variation influenced the HKa-TetraHPRG-tropomyosin binding. All these results indicate that both metal ions are crucial in the interaction between TetraHPRG, tropomyosin and HKa.

## 1. Introduction

The histidine–proline-rich glycoprotein (HPRG/HRG) is a single chain (75 kDa) protein, produced by the liver and present at high concentrations in plasma of vertebrates. It is characterized by a multi domain structure enabling to interact with various ligands, including heparin [1], phospholipids [2], plasminogen [3], fibrinogen [4], immunoglobulin [5], heme [6], and metals [7]. The ability of HPRG to interact simultaneously with various ligands has suggested [8]. It can act as an adaptor molecule in the modulation of the immune, vascular, and coagulation systems, as well as regulating important processes such as cell adhesion, coagulation, fibrinolysis and angiogenesis [2].

The antiangiogenic activity of HPRG has been brought back to the specific interaction of its His/Pro rich domain (H/P) to tropomyosin exposed on the surface of endothelial cells during angiogenic switch. Tropomyosin is part of the actin cytoskeleton and it is present on the surface of proliferating activated endothelial cells during the angiogenic switch, representing a receptor for multiple anti-angiogenic proteins [9], to which belong another evolutionarily and structurally related protein, the high molecular weight kininogen (HKa).

Indeed the HPRG displays a high affinity to tropomyosin, (Kd~2.5 nmol/L) [10,11] and the binding is further favored by mildly acidic conditions (pH < 6.5), such as those observed within a tumor hypoxic region. The unusually high content of histidine residues (pKa, 6.5), (13% of its total amino acid content) [12] confers to the protein a pH sensor activity due to the recognition of negatively charge molecules [12].

HPRG, through its H/P domain, acquires a positive net charge upon exposure to acidic environments, that result crucial in its complex recognition abilities. The H/P domain appears to interact with negatively charged tropomyosin only when the H/P domain displays a total positive charge either through binding to Zn^2+^ via non-charged histidine residues, or by direct protonation of histidine residues induced under mild acidic conditions [10], suggesting a relevant electrostatic contribution to this binding. The H/P domain consists of conserved 12 tandem repeats encompassing the five amino acid sequences (G)HHPH(G). This region has been investigated [11] and the shorter sequence Ac-(HHPHG)_4_-NH_2_ has been considered an effective HPRG mimic system being able to inhibit angiogenesis [13], as well demonstrated. The previous mentioned HKa, also requires a direct binding to tropomyosin to produce antiangiogenic activity, and it is characterized by a domain (D5) that shows a high similarity with H/P of HPRG suggesting analogous functions. In particular, both HKa-D5 and H/P domain of HPRG display a primary sequence rich of positive charges and lack of disulfide bonds that favor the binding to heparin and zinc ions [14]. Indeed, both these domains mediate the binding to endothelium which is in turn favored by elevated concentrations of Zn^2+^ released from activated platelets. The latter increase particularly in number in plasma cancer patients causing thrombotic occlusion of vessels. The levels of free Zn^2+^ in plasma are tightly regulated and are usually too low to promote binding of HPRG to endothelial cells. However during coagulation, the activated platelets release free Zn^2+^ from α-granules [15], and the local concentration of metal ions reaches the levels (approximately 10–50 μmol/L) needed to support the binding of HPRG to tropomyosin on endothelial cells. This interaction is believed essential to mediate the HPRG antiangiogenic effects.

Several investigations suggest that in addition to Zn^2+^ also Cu^2+^ levels dysregulation are related with cancer [16,17]. Furthermore copper is a well-known angiogenic factor [18], which serum concentration is significantly increased in patients affected by cancers including lymphoma, reticulum cell sarcoma, bronchogenic and laryngeal squamous cell carcinomas, cervical, breast, stomach, and lung cancers [19,20]. Noteworthy, the copper levels are related with the stage of the disease and useful to predict relapse and response to therapy [17]. The level of serum copper decreased during periods of remission, sometimes reaching normal levels, and then it rebounded to pre-therapy levels during relapses. It is to note that, unlike copper, the levels of zinc, iron, and selenium are often lower in the serum of cancer patients [21,22,23,24]. In fact, the Cu/Zn, Cu/Fe, and Cu/Se ratios all appear to be better indicators of the presence of cancer than Cu, Zn, Fe, or Se levels alone [21]. Copper is the only metal ions mobilized in endothelial cells during angiogenic processes [25]. This could explain the excess of copper ions found in the extracellular space of tumor cells that use angiogenesis to develop and grow [26].

In this paper, the multiple analogies between copper and zinc, led us to evaluate the potential effect of copper ions in the interaction of the peptide fragment [Ac-(GHHPH)_4_-G-NH_2_] (TetraHPRG) belonging to H/P domain of HPRG, with HKa/tropomyosin.

We used a blocked peptide to N- and C- termini [11] to proper mimic a protein fragment and the role of zinc and copper ions in the interaction with tropomyosin was explored by means of ELISA assay and conformational analysis by circular dichroism (CD). Furthermore, the complex formation of Zn^2+^ TetraHPRG was compared to that of Cu^2+^ by means of potentiometric study [27], to assess the relevance of pH and metal complex species in HKa/Tropomyosin/TetraHPRG binding.

## 2. Results

### 2.1. Tropomyosin/HKa Binding: In Presence of TetraHPRG, Cu^2+^ or Zn^2+^

To unveil the role of copper ions in the interaction between TetraHPRG and tropomyosin, an ELISA assay was carried out. In the experimental procedure, the plate was coated by human cardiac tropomyosin, while HKa* and the TetraHPRG were added in buffer as competitive agents. Differently from a similar assay previously reported [11], here we verified the effect of some experimental variables, such as the pH and order of addition of reagents (see Methods section) that deeply influenced the amounts of HKa* detected.

As first step, performing a dose–response curve, the saturation concentration of biotinilated–HKa (HKa*) was determined. The minimal concentration of HKa*, able to produce the highest signal obtainable by its binding to tropomyosin, was 80 nM.

Successively, the ability of TetraHPRG to compete with HKa* in the binding with tropomyosin has been tested through the coincubation of TetraHPRG, at different concentrations and maintaining HKa* concentration set to 80 nM. In the concentration range investigated (5–50 µM), the TetraHPRG induced a mild reduction of the levels of HKa* bound to tropomyosin, confirming that the peptide, although at different orders of magnitude, has affinity to tropomyosin and may compete with HKa*.

On the contrary, the shorter sequence i.e., the hexapeptide, encompassing only one repeat unit Ac-GHHPHG-NH_2_, has no effect, even at 100 µM, that correspond to 25 µM concentration of TetraHPRG.

**Figure 1:**
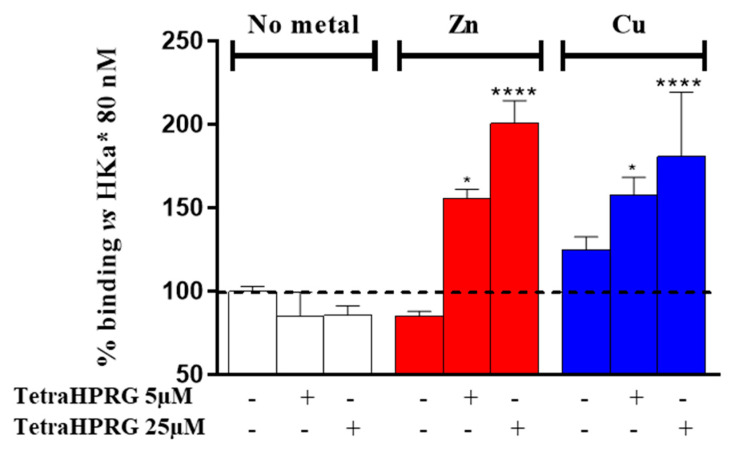
Binding rate of HKa* at 80 nM in MOPS pH 7.5. Dose response effect of tetra repeat in coincubation in presence or Zn^2+^ or Cu^2+^ (10 µM). Data are the mean ± SEM of three different experiments performed in triplicate. Statistically significant differences are indicated with * = *p* < 0.05 and **** = *p* < 0.0001 vs. HKa 80 nM (One-Way ANOVA + Dunnett’s test).

The metal effect was evaluated using copper or zinc ions at 10 µM. As showed in Figure 1, in the presence of both metal ions, the TetraHPRG resulted to be not competitive in the binding to tropomyosin; on the contrary, we could notice an increase of HKa* bound. Therefore, the dose–response trend observed in Figure 1 clearly indicates, that the TetraHPRG, in the used experimental conditions, promoted the tropomyosin-HKa* interaction, suggesting the presence of a ternary system due to the interaction among proteins, peptide and metal ions.

Furthermore, it should be highlighted that in absence of TetraHPRG, the behavior of zinc and copper is opposite; i.e., the Zn^2+^ slightly reduced the amount of HKa* bound to tropomyosin, on the contrary, the Cu^2+^ slightly increased it.

A hypothesis is that the metal ions differently affected the conformational features of molecules involved in the tropomyosin–TetraHPRG–HKa* interaction. Previously, it was reported that the binding of zinc to domain D5 (HKa domain 5) induced a conformational change in the entire HKa molecule [28], unlike other metal ions, as well as Cu^2+^. Thus, CD measurements were carried out to assess if a similar effect is observed for the investigated systems.

CD is largely used to study the secondary structure of proteins and peptides [29,30,31,32] with the advantage to use low sample concentration and give information also on the effect of added ligands.

**Figure 2:**
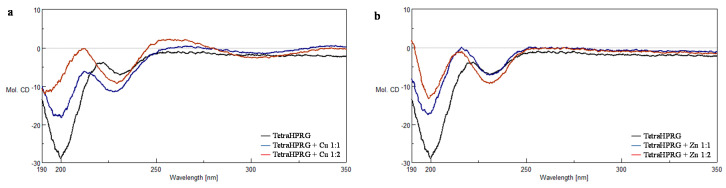
Circular dichroism (CD) spectra of (**a**) copper (II) and (**b**) zinc (II) complexes with TetraHPRG in water (pH = 7.5), at different mol equivalent of metal to ligand molar ratio is 1:1 and 2:1; [L] = 1 × 10^−5^ M).

First of all, the effect of each metal ions addition to peptide (named binary systems) conformation was explored (Figure 2a,b). Taking into account the high number of proline residues, the CD spectrum features of TetraHPRG may result from an equilibrium among PPII, random coil and β-turn conformations [33]. The presence of PPII conformation was supported by the CD spectrum that showed a broad minimum at 199 nm and a slightly negative at about 233 nm [34,35]. Interestingly, the addition of copper sulphate induced changes in the peptide dichroic signals, while the band at 199 nm decreased, the band at 233 nm inversely increased (Figure 2a). The conformational modifications are more evident at increasing amount of copper ions equivalent.

Moreover, the peptide/copper(II) spectra showed the appearance of a signal at about 260 nm, that can be assigned to the charge transfer band N_im_→Cu^2+^, and a weak negative band around 300 nm, hallmark of the charge transfer transitions of deprotonated amide nitrogen atoms toward metal ions, N^−^_amide_→Cu^2+^ [36]. These results showed the involvement of the histidine residues in the copper coordination environment.

The CD spectra TetraHPRG carried out after the addition of zinc(II) are reported in Figure 2b. The perturbation of the ligand conformation due to the addition of the metal ions was different than that observed for the analogous complex with copper, suggesting a more structured conformation of peptide induced by zinc binding.

The speciation and stability constant values for copper complexes formed with TetraHPRG have been reported [27]. Therefore, potentiometric titrations were carried out to determine zinc complex species formation with TetraHPRG and to compare with those obtained for copper ions.

**Figure 3:**
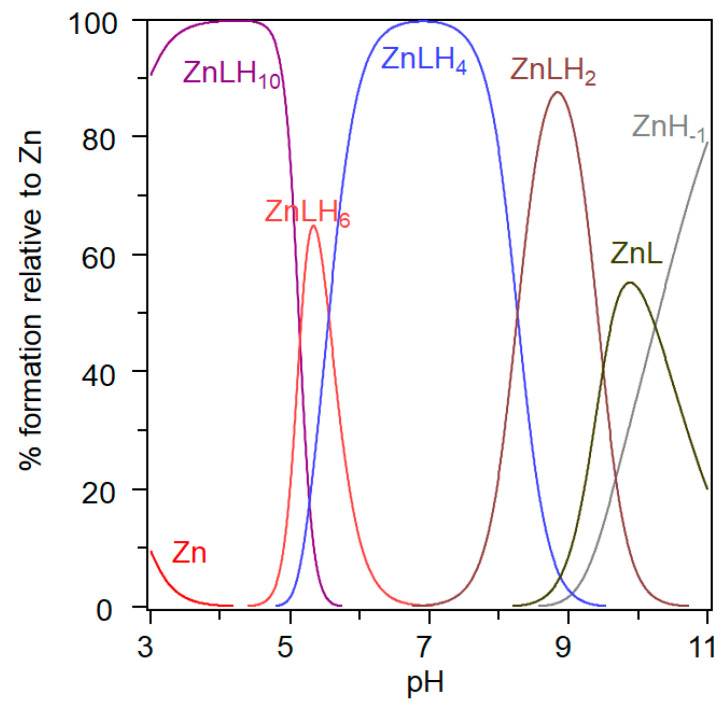
Species distribution diagram for the Zn^2+^ complexes with TetraHPRG at 0.5:1 (M:L) molar ratio. [Zn^2+^] = 1 × 10^−3^ M.

The protonation constants are reported in Table S1. Because of the high number of histidine residues in the primary sequence not all protonation constants were determined [27]. The differences between the pK values are small and the deprotonation processes take place in overlapping equilibria. Therefore, each protonation constant ha to be considered as a macro-constant and not assigned to a specific histidine residue [37,38]. The obtained pK values are in line with those reported for poly-histidine peptides [39]. The stability constant values of zinc complexes species are listed in Table 1 and the distribution diagram is reported in Figure 3. Measurements were carried out at different metal to ligand molar ratios ranging from 0.5:1 to 2:1 (see supporting materials). Mononuclear complex species are present only at 0.5:1 metal to ligand molar ratio. Increasing the metal to ligand molar dinuclear complex species were formed. (see Figure S1).

During potentiometric measurements of Zn^2+^ complexes, His residues deprotonate in a short pH range and probably some in pairs in an overlapping mode due to closest values of their pK value [27].

The predominant zinc complex species formed in the pH range 6–8 is ZnLH_4_. Taking into account the deprotonation steps and the stability constant value it is possible to suggest the coordination of four imidazole nitrogen atoms to zinc ion [40,41,42,43,44]. In the [ZnLH_2_] complex species the further deprotonation steps most probably come from other histidine residues which do not participate in binding even though the involving of amide nitrogen peptide backbone has been reported [45].

Increasing the equivalent of zinc ions, dinuclear species were detected. However, either at 1:1 or at 2:1 metal to ligand molar ratio, precipitation phenomenon occurred starting from pH 5, so it was not possible to determine with sufficient accuracy the stability constant values of complex species containing two zinc ions (see supplementary Figure S1).

**Figure 4:**
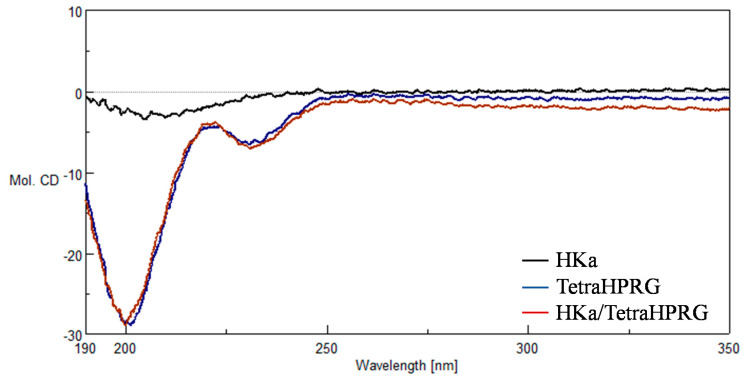
CD spectra Hka, TetraHPRG, and Hka/TetraHPRG in water (pH = 7.5), [L] = 1 × 10^−5^ M.

The CD spectrum of the HKa/TetraHPRG binary system is reported in Figure 4. The HKa protein showed a weaker intensity of dichroic signals respect to those of peptide alone. No significative conformational differences were observed when TetraHPRG was added to HKa, indicating that they did not interact significantly, in agreement with ELISA assay results (see Figure 2).

**Figure 5:**
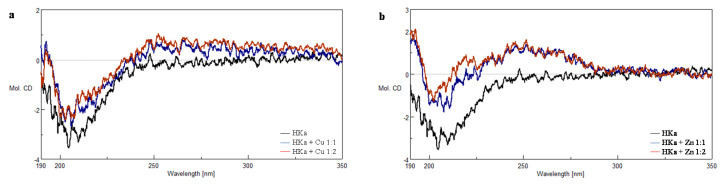
CD spectra of (**a**) copper(II) and (**b**) zinc(II) complexes with Hka in water (pH = 7.5), at different mol equivalent of metal (metal to ligand molar ratio is 1:1 and 2:1; [L] = 1 × 10^−5^ M).

Both copper (II) and zinc (II) induced a reduction of dichroic signal of the HKa, although a prevalently random coil structure was maintained, as showed in Figure 5. No significant differences were observed increasing the metal ions equivalent at 2:1 compared to 1:1 spectra, but on the whole the zinc (II) addition was more effective than copper (II) in affecting the conformation of HKa.

**Figure 6:**
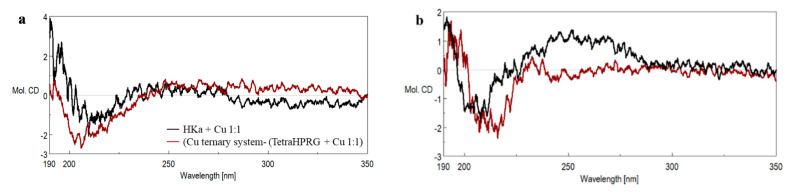
CD difference spectra of the (**a**) Hka/TetraHPRG/ Cu^2+^ ternary system minus CD spectra from TetraHPRG/Cu^2+^ systems and CD spectra of the Hka/ Cu^2+^ at 1:1 metal to ligand ratios and (**b**) CD difference spectra of the Hka/TetraHPRG/Zn^2+^ ternary system minus normalized CD spectra from TetraHPRG/Zn^2+^ systems and CD spectra of the Hka/Zn^2+^ at 1:1 metal to ligand ratios.

The conformational analysis of ternary systems showed the presence of two negative bands at about 195 nM and 225 nM and the spectra were very similar to that of TetraHPRG/ Cu^2+^ binary systems. Taking into account the strong difference in the dichroic signals between HKa and TetraHPRG, the signals of the binary systems, TetraHPRG/Cu^2+^ and TetraHPRG/Zn^2+^, were subtracted to those of ternary systems, HKa/TetraHPRG/Cu^2+^ and HKa/TetraHPRG/Zn^2+^, respectively (Figures S2 and S3). The difference spectra together with binary system HKa/Cu^2+^ spectra are reported in Figure 6a. As observed, the differences spectra are not superimposable to that of binary systems, suggesting a dichroic contribute due to formation of an interaction between protein, peptide, and copper.

The resultant difference between the spectra HKa/TetraHPRG/Zn^2+^ and TetraHPRG/Zn^2+^ showed a CD profile completely different to that of HKa/Zn^2+^ spectrum, evidencing a positive band at about 220 nM (Figure 6b). Such behavior suggested that the intensity of dichroic signals of CD spectra was not only due only to the sum of the single contribute of HKa/Zn^2+^ and TetraHPRG/Zn^2+^ binary systems, but, as well as for copper, also to a contribute of the contemporary interaction between HKa, TetraHPRG and zinc. Furthermore, the interaction with the copper and zinc influenced the HKa conformational profile in a different way, suggesting a different binding modes in the two ternary systems.

### 2.2. pH Effect

The TetraHPRG is a sequence of the repeats rich in Pro and His residues [11] which may have a key role in the antiagiogenic activity [46]. Taking into account the pKa values of histidine, the binding properties of this domain are strongly influenced by electrostatic forces [47] that can be also influenced by the presence of metal ions, ionic strength and pH value.

**Figure 7:**
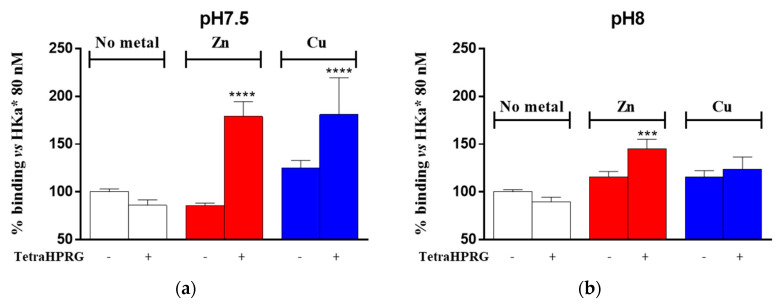
Percentage of HKa* binding to tropomyosin at different pH. HKa* (80 nM) in MOPS pH 7.5 (**a**) or pH 8.0 (**b**), in presence or not of 25 uM of TetraHPRG and in presence or not of CuSO_4_ or ZnCl_2_ (10 µM). Data are the mean ± SEM of three different experiments performed in triplicate. Statistically significant differences are indicated with *** = *p* < 0.001 and **** = *p* < 0.0001 vs. HKa 80 nM (One-Way ANOVA + Dunnett’s test).

To verify that tropomyosin binding assay has the sufficient sensitivity to appreciate the electrostatic contribution into interaction HKa*/TetraHPRG/tropomyosin metal-mediated, we performed the ELISA assay at pH increasing only half unit of pH respect to standard experimental settings (pH 7.5), in coincubation conditions. While at pH 7.5 the metal promoted the binding of TetraHPRG with HKa* (as reported in Figure 2), at pH 8.0 the metal reduced the interaction (Figure 7). As reported by Donate et al. [11], the H/P domain appeared to interact with tropomyosin, negatively charged, only when the H/P domain acquired a positive charge either, through binding Zn^2+^ via neutral His residues or by direct protonation of histidines induced under mild acidic conditions [14]; this suggests a large electrostatic contribution to the binding. The results reported in Figure 7, confirmed the involvement of histidine residues in the interaction between TetraHPRG and tropomyosin mediated by zinc(II) and suggested a similar behavior also for copper(II) ion.

### 2.3. TetraHPRG Effect in “Pre-Treatment”

**Scheme 1:**
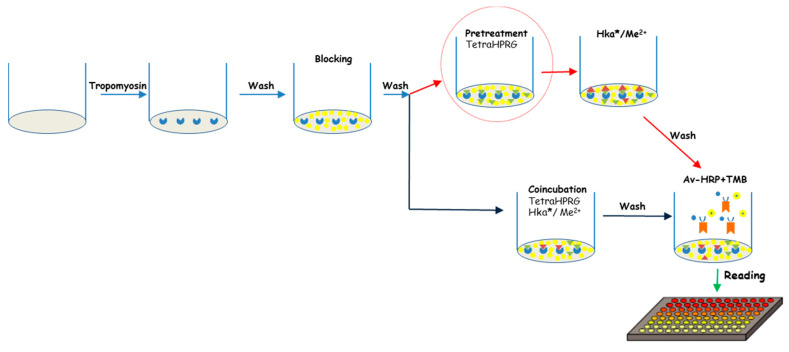
ELISA assay steps in conditions of coincubation (down) and pre-treatment (up). HKa* (Kininogen activated), Me^2+^ (Cu^2+^ or Zn^2+^), Av-HRP (avidin conjugated with horseradish peroxidase), TMB (3,3-5,5-tetramethylbenzidine).

To better elucidate the effect of TetraHPRG on HKa* binding to tropomyosin, the order of molecules addition was modified, depositing the peptide directly on the plate with tropomyosin, after the blocking step and before the incubation step of HKa*. This modality that we defined as pre-treatment (see Scheme 1) allowed to isolate the single components involved in the binding phenomena, respect to the coincubation where all molecules are mixed together.

**Figure 8:**
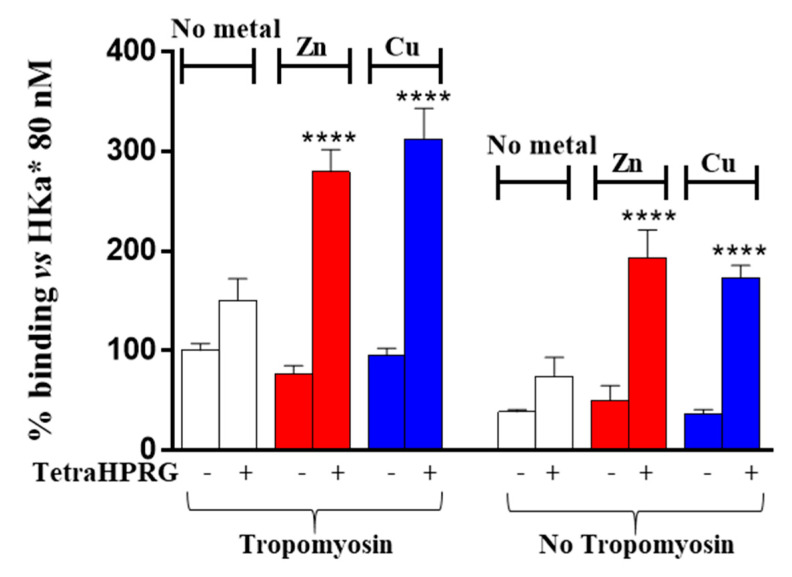
% Binding of HKa* to plate previously coated with tropomyosin (left group) or not (right). After tropomyosin the plate has been blocked with milk 5% and the pretreated with TetraHPRG at 25 µM before to the addition of HKa* (80 nM) in MOPS pH 7.5 in presence or not of CuSO_4_ or ZnCl_2_ (10 µM). Data are the mean ± SEM of three different experiments performed in triplicate. Statistically significant differences are indicated with **** = *p* <0.0001 vs. HKa 80 nM (One-Way ANOVA + Dunnett’s test).

As first evidence of the modifying ELISA conditions in pre-treatment and in absence of metals, the TetraHPRG at 25 µM overturns its competitive effect, and surprisingly, the levels of HKa* bound were increased of about + 50%. This effect was positively strengthened by the presence of Cu^2+^ or Zn^2+^ ions, as can be observed by the high intensity of HKa* detected (Figure 8, left group); this result may be due to a favorite formation of a ternary complex tropomyosin-TetraHPRG-HKa*, whose existence seems to be supported by CD results.

Successively, observing that the TetraHPRG deposition on a plate (in the pretreatment modality) increased significantly the adhesivity to HKa*, we decided to evaluate if this adhesiveness was mediated by the tropomyosin or by the “blocked surface”. For this purpose, assays in absence and in presence of tropomyosin, using milk protein as blocking step, were performed. Although in the presence of tropomyosin the levels of HKa* bound were higher (about double) respect to corresponding samples in absence of tropomyosin, the binding trend was comparable (Figure 8, right group). The addition of each metal ions induced a similar increase in the percentage of HKa* bound, confirming that both zinc and copper ions were crucial in the interaction between TetraHPRG and HKa*.

## 3. Discussion

The HPRG protein is a well-known angiogenic modulator, although the mechanisms of its anti-angiogenic activity are not fully understood. An hypothesis, provided by in vitro and in vivo studies [11], points to the binding of H/P reach domain of HPRG with tropomyosin, a protein normally found inside cell where stabilizes actin filaments of cytoskeleton and expressed by endothelial cell during angiogenic process. This interaction activates anti-angiogenic signaling transduction pathways. In a similar way, the high molecular weight kininogen (HKa) inhibits angiogenesis [13], involving a direct binding to tropomyosin. The HKa-D5 (HKa domain 5) and the H/P domain of HPRG, resulted to have a similar binding to heparin and metals, and an abundance of positive charges. The evidence that H/P peptide blocks HKa binding to tropomyosin support that HPRG and HKa share the same receptor. Therefore, a comparison between HKa and the peptide TetraHPRG ability to bind tropomyosin could provide useful information, in particular on the role of copper and zinc ions in the tumor microenvironment. The effect of zinc binding to ternary system Hka-TetraHPRG-tropomyosin has been extensively studied and although copper shares with zinc similar coordination binding environment, at the best of our knowledge, no data have been reported about the role of copper in such system.

TetraHPRG, (Ac-(HHPHG)_4_-NH_2_) a blocked tetramer at N- and C-termini was synthesized as an effective HPRG mimic system, able to compete with the whole protein in binding to the HUVEC tropomyosin and heparin sulfate proteoglycan receptors [11]. Differently from assays reported in literature, that used chicken tropomyosin, in this work human tropomyosin was employed.

In experimental conditions of the present work, ELISA assays show that, at pH 7.5, zinc ions reduced the HKa-tropomyosin binding, while copper increased it. CD spectra of HKa after the addition of metal ions show that zinc induces a major perturbation of HKa conformation. This is in agreement with the conformational changes reported for the entire HKa molecule due to the zinc binding to the HKa-D5 [28] domain. Such effect has not been reported for other metals and in particular for copper.

The conformation of TetraHPRG is affected by both zinc and copper ion. Moreover, the potentiometric titrations suggested that peptide binds tightly zinc and the main species at physiological pH, is ZnLH_4_, in which the metal coordination environment involves four imidazole nitrogen atoms. Increasing the pH, the deprotonation of amide nitrogen atoms should also occur. However, this is suggested only by the different p*K*a values for the zinc complex in comparison to the free ligand, as spectroscopic data are not available for d^10^ electronic configuration of Zn^2+^.

CD conformational analysis showed that TetraHPRG spectrum features underlined an equilibrium between PPII and random coil conformations. The TetraHPRG peptide is able to partially maintain the conformation PPII observed for this domain in the whole protein [27]. The addition of metal ions affected the peptide conformation, suggesting the presence of a turn conformation induced by copper or zinc binding. The CD spectra bands at 260 nM and 300 nM, typical of ligand to copper(II) charge transfer of imidazole and amide nitrogen atoms, confirmed the coordination environment of metal as suggested by potentiometric measurements at physiological pH [27].

In the presence of TetraHPRG, copper ions, as well as zinc ions, promote the binding between HKa and tropomyosin. Furthermore, this phenomenon is confirmed by the ternary system formation TetraHPRG-HKa-metal (zinc or copper), whose existence is corroborated by conformational changes induced by metal coordination [48,49]. In fact, the CD spectrum of a mixture of HKa and TetraHPRG, is the result sum of these separate components, suggesting the absence of a direct interaction. Interestingly, the addition of metal ions resulted into significant variations in the spectra profiles not simply attributable to the formation of the metal-TetraHPRG complex. In differences spectra, the appearance of new bands, suggests the formation of a ternary complex system between HKa, peptide and metal. This can explain the increase in the binding to tropomyosin of HKa in the contemporary presence of TetraHPRG and metal ions. In ternary systems the interaction of zinc is more effective than copper ions suggesting a potential different coordination modes.

Interestingly, a slight pH variation influenced the HKa-TetraHPRG-tropomyosin binding. The activity of TetraHPRG is related to the presence of histidine residues for both, metal binding and charge effects. At pH 8.0 the amount of [CuL] increase, and in this species there are two deprotonated amide nitrogen atoms, that neutralizes the positive charges of metal. These combined effects may explain the drastic reduction of HKa* bound to tropomyosin at pH 8.0 compared to pH 7.5 and confirm the involvement of charged histidine residues in the interaction between TetraHPRG and tropomyosin as suggested in a previous work [11].

Intriguingly, in pre-treatment condition the TetraHPRG overturns its competitive effect, and the levels of HKa* bound increase of about +50%. This effect is positively strengthened by the presence of Cu^2+^ or Zn^2+^ ions.

This condition can also be attributable to a favorite formation of a ternary complex tropomyosin-TetraHPRG-HKa*. Moreover, we found that although in the presence of tropomyosin the levels of HKa* bound were higher respect to corresponding samples in absence of tropomyosin, the pattern of binding was comparable. In a similar way, the addition of both metal ions induced an increase of percentage of HKa* bound, confirming that metal ions were crucial in the binding between TetraHPRG and HKa*.

The experimental conditions used in this study pinpoints the property of the interaction between TetraHPRG/HKa with tropomyosin, highlighting how it may be strongly influenced by the presence of metal ions, ionic strength, and pH value. Specifically, TetraHPRG and HKa antiangiogenic property is enhanced by the presence of metal ions, promoting the formation of a ternary complex among proteins, peptide and metal ions. This effect suggests that copper, like zinc, may mediate the binding of HPRG to the tropomyosin exposed on the endothelial cell surface and modulate its antiangiogenic activity. Thus, the use of TetraHPRG could take advantage of the high copper level typical of tumor microenvironment.

## 4. Materials and Methods

### 4.1. Proteins, Peptides, and Materials

Human cardiac tropomyosin was purchased from Alpha Diagnostics; HKa was obtained from Enzyme Research Laboratories (Swansea, UK). HKa was biotinylated using EZ-Link Sulfo-NHS-LC-biotin (Thermo, Waltham, Massachusetts, USA) following the manufacturer’s instructions.

The peptide [Ac-(GHHPH)_4_-G-NH_2_] (TetraHPRG) has been synthesized in the N-acetylated and C-amidated form to mimic more properly its character of a protein internal fragment. The synthesis was carried out by means of solid phase peptide synthesis as previously reported [27]. Avidin-horseradish peroxidase (Av-HRP) was acquired from Sigma (Saint Louis, Missouri, USA) and 3,3-5,5-tetramethylbenzidine (TMB) was purchased from Enzo life (Farmingdale, New York, USA).

### 4.2. ELISA Assay

Human cardiac tropomyosin (Alpha Diagnostics, San Antonio, TX, USA) was dissolved in TBS (Tris 50 mM pH 7.5 + NaCl 140 mM) and 200 ng were added/well in a 96-well high binding polystyrene assay plate (Santa Cruz, Dallas, Texas, USA), and then incubated for 6 h at room temperature. The plate was washed with TBS-T (TBS + Tween20 0.05%) and blocked with not fat milk (5%) in TBS, overnight. Then, 80 nM biotinilated HKa (HKa*) was added in the presence of 10 µM metal ions (ZnCl_2_/CuSO_4_) together with TetraHPRG 25 µM, in MOPS 10 mM at pH 7.5 and 8.0, and incubated for 2 h at RT. The unbound HKa* was then removed washing five times with the TBS-T buffer. Then bound HKa* was detected using avidin-horseradish peroxidase (Av-HRP) and the chromogenic substrate 3,3-5,5-tetramethylbenzidine (TMB) at 450nM.

### 4.3. CD Spectroscopy

CD spectra were obtained at 25 °C under a constant flow of nitrogen on a Jasco model 1500 spectropolarimeter at a scan rate of 50 nM min^−1^ and a resolution of 0.1 nM. The path length was 1 cm, in the 190–350 nM range. The spectra were recorded as an average of 10 or 20 scans (data points 1601). Calibration of the instrument was performed with a 0.06% solution of ammonium camphorsulfonate in water. All the solutions were freshly prepared using MOPS 10 mM at pH 7.5. CD spectra were acquired by using 1.0 × 10^−5^ M peptide concentration at different metal to ligand ratios 1:1 and 2:1 in the presence or not of 1.3 × 10^−8^ M kininogen (HKa). The results are reported as Δε (molar dichroic coefficient) in M^−1^.

### 4.4. Potentiometric Titrations

Potentiometric titrations were performed on automatic instrument Titrando 905 using a combined glass-Ag/AgCl electrode (Metrohm, Herisau, Switzerland). Aqueous solutions of samples (2.0 mL) were kept under an argon atmosphere and at 298 K by means of a thermostat. Other details on the electrode calibration have been previously reported [50]. In all solutions, the ionic strength was fixed at 0.1M by adding KNO_3_. KOH 0.1 M was used to titrate solutions containing either the peptides only or the peptide with Zn^2+^. The peptide concentrations employed were 1 × 10^−3^ M. At least three independent titrations were performed. Starting pH value was adjusted to 2.4 by adding HNO3 0.2 m and measurements were carried out up to pH 8.5. Metal ion/ligand ratios between 0.9:1 and 2.2:1 were employed. The data were analyzed by using HYPERQUAD 2003 program [51] and species distribution as a function of pH was obtained by using Hyss program [52].

### 4.5. Statistical Analysis

Graph-Pad Prism (Version 7.00) was used for data analysis and graphic presentation. Data were reported as the mean ± SEM of at least three or two different experiments. Statistical analyses were performed using a one-way ANOVA study followed by the Dunnett test for repeated measurements. Differences were considered statistically significant when *p* < 0.05.

## Figures and Tables

**Table 1:** Stability constants (log β) and pK values of the Zn^2+^ complexes with TetraHPRG (L) (T = 298 K, I = 0.1 M KNO_3_) ^1^.

Species	Log Beta
1 1 10	71.65 (4)
1 1 6	51.08 (6)
1 1 4	39.98 (4)
1 1 2	23.44 (5)
1 1 0	4.45 (6)

^1^ Standard deviations (3σ values) are in parentheses. Charges are omitted for clarity.

## Supplementary Materials

The following are available online at https://www.mdpi.com/1422-0067/21/24/9343/s1, Table S1: Protonation constants of TetraHPRG; Figure S1: Species distribution diagram for the Zn^2+^ complexes with TetraHPRG with M/L molar ratio 2:1.; Figure S2: CD spectra of copper(II) complexes with Hka, TetraHPRG and of ternary systems (Hka/TetraHPRG/Cu^2+^:; Figure S3: CD spectra of zinc(II) complexes with Hka, TetraHPRG and ternary systems (Hka/TetraHPRG/Zn(II)).
