# Supplementary material for: The Role of Copper (II) on Kininogen Binding to Tropomyosin in the Presence of a Histidine–Proline-Rich Peptide
Source: Int J Mol Sci. 2020 Dec 8;21(24):9343. doi: 10.3390/ijms21249343 (PMC7762548; doi:10.3390/ijms21249343)
Supplement: Supplementary file 1 [file ijms-21-09343-s001.pdf]

International Journal of *Molecular Sciences*

MDPI

Supplementary Materials

Copper Affects Binding to Tropomyosin of Peptides Derived from Histidine-Proline-Rich Glycoprotein

Anna Maria Santoro [1], Stefania Zimbone [1], Antonio Magrì [1], Diego La Mendola [2,*] and Giulia Grasso [1,*]

[1] CNR Istituto di Cristallografia Sede Secondaria di Catania, Via Gaifami 18, 95126 Catania, Italy; annamaria.santoro@cnr.it (A.M.S.); stefania_zimbone@libero.it (S.Z.); antonio.magrì@cnr.it;

[2] Dipartimento di Farmacia, Università di Pisa, Via Bonanno Pisano 6, 56126 Pisa, Italy;

* Correspondence: lamendola@farm.unipi.it (D.L.); giulia.grasso@cnr.it (G.G.)

Table S1. Protonation constants (log β) TetraHPRG (L) (T = 298 K)[a].

Species	logβ	pK
LH	7.49 (5)	7.49
LH_2	14.65 (4)	7.16
LH_3	21.29 (9)	6.91
LH_4	28.20 (2)	6.63
LH_6	40.88 (2)	(6.34 x 2)
LH_8	52.52 (3)	(5.83 x 2)
LH_9	57.96 (6)	5.44
LH_{10}	63.24 (6)	5.28
LH_{11}	68.59 (3)	5.35
LH_{12}	73.15 (2)	4.55

[a] Standard deviation (3σ values) are given in parenthesis. The charges are omitted for clarity [27].

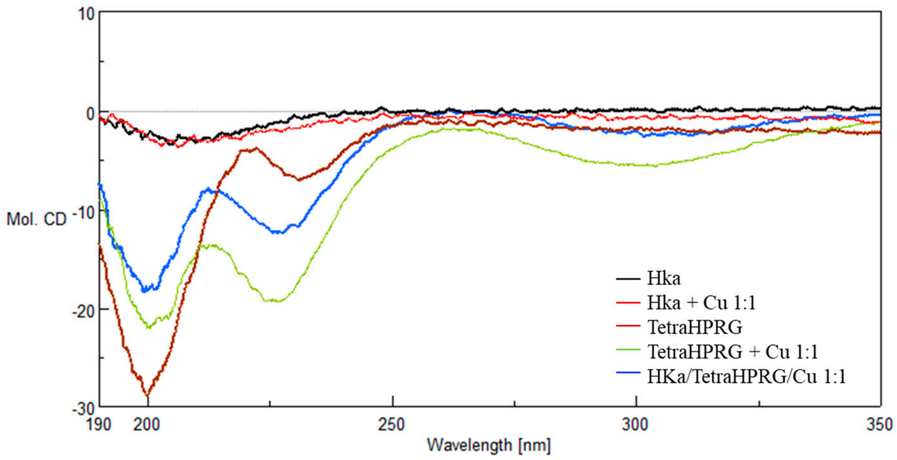

Figure S1. CD spectra of a)copper(II) complexes with Hka, TetraHPRG and ternary systems (Hka/TetraHPRG/Cu^{2+} in water (pH=7), at different mol equivalent of metal (metal to ligand molar ratio is 1:1; [L]=1×10^{-5} M).

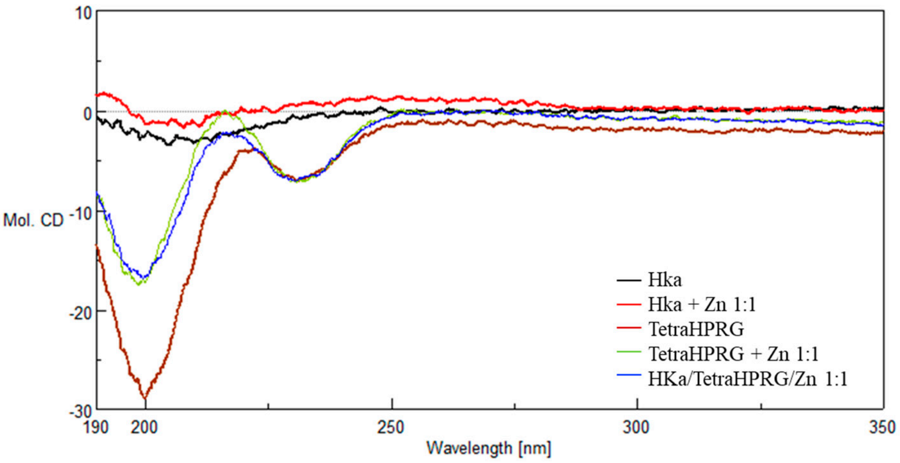

Figure S2. CD spectra of a)copper (II) complexes with Hka, TetraHPRG and ternary systems (Hka/TetraHPRG/Cu (II)) in water (pH=7), at different mol equivalent of metal (metal to ligand molar ratio is 1:1; [L]=1×10^{-5} M).

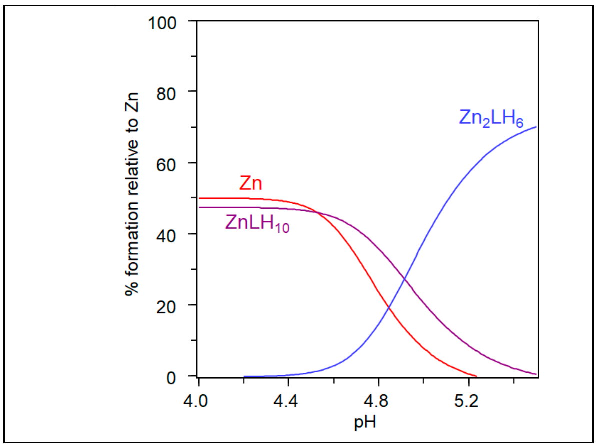

Figure S3. Species distribution diagram for the Zn^{2+} complexes with TetraHPRG with M/L molar ratio from 1:1 to 2:1. [Zn^{2+}] = 1 × 10^{-3} M.
